# Benefits and risks of drug combination therapy for diabetes mellitus and its complications: a comprehensive review

**DOI:** 10.3389/fendo.2023.1301093

**Published:** 2023-12-19

**Authors:** Xueqin Xie, Changchun Wu, Yuduo Hao, Tianyu Wang, Yuhe Yang, Peiling Cai, Yang Zhang, Jian Huang, Kejun Deng, Dan Yan, Hao Lin

**Affiliations:** ^1^ Center for Informational Biology, School of Life Science and Technology, University of Electronic Science and Technology of China, Chengdu, China; ^2^ School of Basic Medical Sciences, Chengdu University, Chengdu, China; ^3^ Innovative Institute of Chinese Medicine and Pharmacy, Academy for Interdiscipline, Chengdu University of Traditional Chinese Medicine, Chengdu, China; ^4^ Beijing Friendship Hospital, Capital Medical University, Beijing, China

**Keywords:** diabetes, diabetic complications, diabetic management, drug combination therapy, blood glucose control, personalized medicine

## Abstract

Diabetes is a chronic metabolic disease, and its therapeutic goals focus on the effective management of blood glucose and various complications. Drug combination therapy has emerged as a comprehensive treatment approach for diabetes. An increasing number of studies have shown that, compared with monotherapy, combination therapy can bring significant clinical benefits while controlling blood glucose, weight, and blood pressure, as well as mitigating damage from certain complications and delaying their progression in diabetes, including both type 1 diabetes (T1D), type 2 diabetes (T2D) and related complications. This evidence provides strong support for the recommendation of combination therapy for diabetes and highlights the importance of combined treatment. In this review, we first provided a brief overview of the phenotype and pathogenesis of diabetes and discussed several conventional anti-diabetic medications currently used for the treatment of diabetes. We then reviewed several clinical trials and pre-clinical animal experiments on T1D, T2D, and their common complications to evaluate the efficacy and safety of different classes of drug combinations. In general, combination therapy plays a pivotal role in the management of diabetes. Integrating the effectiveness of multiple drugs enables more comprehensive and effective control of blood glucose without increasing the risk of hypoglycemia or other serious adverse events. However, specific treatment regimens should be tailored to individual patients and implemented under the guidance of healthcare professionals.

## 1 Introduction

**Figure 1:**
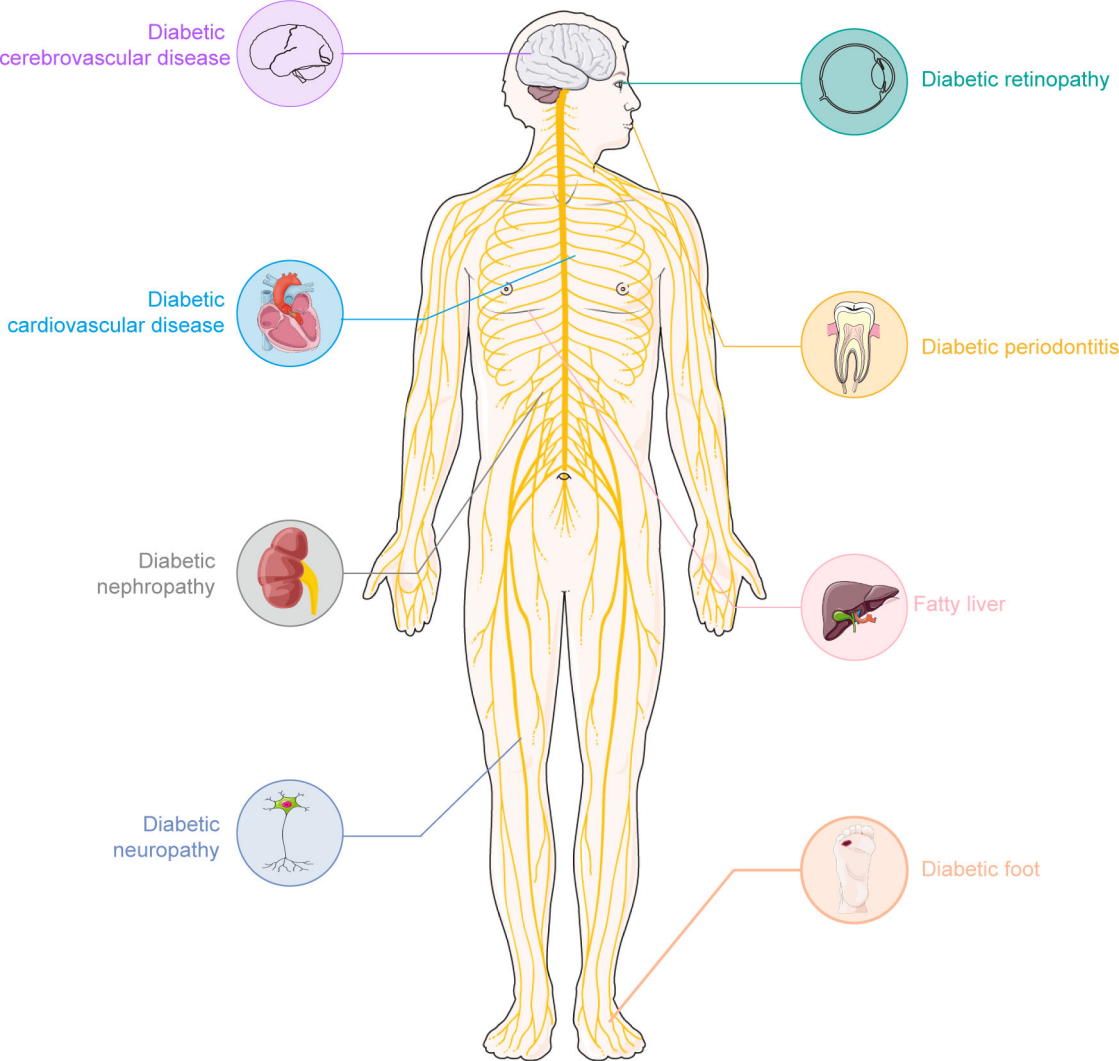
The common complication of diabetes. Diabetes is a chronic disease. Long-term hyperglycemia could lead to many complications. Some common complications of diabetes mainly include: cardiovascular disease, diabetic retinopathy, diabetic neuropathy, diabetic nephropathy, diabetic foot, etc. These complications seriously affect the quality of life in individuals with diabetes. A clearly understanding of these complications and finding appropriate medication will promote the well-being of diabetic patients worldwide.

Diabetes mellitus (DM) is a metabolic disease characterized by hyperglycemia. From the perspective of etiology, it can be mainly divided into two categories: type 1 diabetes (T1D) which is caused by the autoimmune destruction of insulin-producing β cells, and type 2 diabetes (T2D) that is characterized by impaired insulin sensitivity and insulin resistance (1–5). The pandemic of DM has brought a serious public health challenge to the globe (6, 7). It is estimated that by 2045, more than 693 million individuals worldwide will live with diabetes (8). More importantly, DM is a systemic syndrome that impacts nearly all tissues in the human body. According to the different tissues it affects, diabetes has many serious complications (**Figure 1**), including kidney disorders (9), cardiovascular diseases (10), peripheral neuropathy (11), etc.

Due to the complicated pathological progress of diabetes, particularly in the case of T2D, the normal function of β cells will gradually decline with the prolongation of disease course (12). In this case, monotherapy may not achieve the desired effect of blood glucose control in some specific patients. As demonstrated by the UKPDS study, the proportion of patients maintaining glycated hemoglobin A1c (HbA1c) levels < 7% by monotherapy has been decreasing year by year during the follow-up period (13), suggesting that the monotherapy may not be able to effectively control hyperglycemia in the long term. Therefore, an increasing number of clinicians are embracing drug combination therapy (14). Drug combination therapy is a medical strategy that involves the simultaneous administration of two or more drugs, with the aim of achieving a more pronounced therapeutic effect than using a single drug alone. The mechanism of action in drug combination therapy is multifaceted, encompassing synergistic effects, complementary effects, antagonizing resistance, and more. This comprehensive approach contributes to the effectiveness of the treatment. In this review, we systematically assessed the efficacy and safety of drug combination therapy for diabetes and the associated complications based on existing studies so as to provide insights and guidance for clinicians to choose appropriate drug combinations for effective treatment in clinical practice. Based on the existing clinical study, we primarily focus on the seven common classes of antidiabetic medications (**Table 1**), analyzing their potential combinations for the treatment of diabetes and its complications. It is worth noting that, in contrast to clinical cohort studies on dual-drug combination therapy, the majority of evidence supporting multi-drug combination therapy primarily originates from preclinical studies conducted at the animal level. Despite the growing interest and potential in multi-drug approaches, their translation into clinical practice necessitates further exploration and validation through well-designed clinical trials.

**Table 1:** The commonly used classes and some examples of glucose-lowering drugs.

Pharmacological class	Mode of action	Example drug
Biguanides	AMPK activation	Metformin
Sulphonylureas	K(ATP) channel blocking	Glimepiride
		Glibenclamide
		Gliclazide
		Glipizide
SGLT2 inhibitors	SGLT2 inhibition	Canagliflozin
		Dapagliflozin
		Empagliflozin
		Janagliflozin
DPP-4 inhibitors	DPP-4 inhibition	Vildagliptin
		Sitagliptin
		Gemigliptin
		Linagliptin
		Saxagliptin
		Alogliptin
GLP-1 receptor agonists	Incretin effect	Lixisenatide
		Exenatide
		Liraglutide
		Semaglutide
Alpha glucosidase inhibitors	Alpha glucosidase inhibition	Miglitol
		Acarbose
		Voglibose
Thiazolidinediones (TZDs)	PPARγ activation	Pioglitazone
		Rosiglitazone

## 2 Pathogenesis of diabetes

### 2.1 T1D

**Figure 2:**
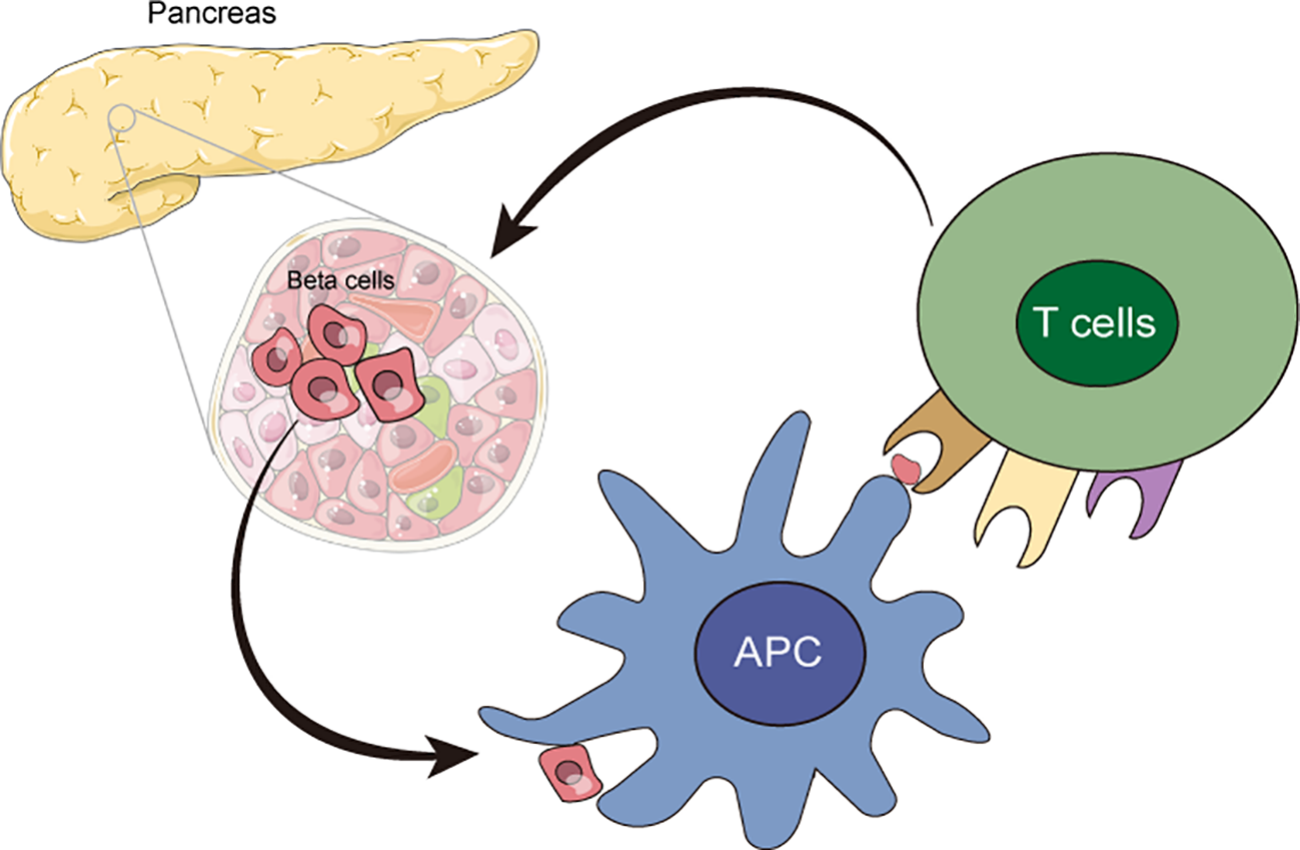
The autoimmune responses in T1D. Autoreactive T cells, including CD4 and CD8 cells, mistakenly recognized insulin-producing β cells as foreign invaders and initiated attacks to β cells, which resulted in the loss and destruction of β cells.

T1D is a chronic autoimmune disease in which the immune system mistakenly recognizes insulin-producing β cells as foreign invaders and initiates an attack to β cells (15). Autoreactive T cells, including CD4 and CD8 cells, have been identified as key contributors to the destruction of β cells (16, 17), resulting in the severe loss of β cells, insulin deficiency, and sustained hyperglycemia (**Figure 2**). Its mechanisms involve innate and adaptive immune responses that might be triggered by the interaction of genetic and environmental factors (18). Genetic factors play a critical role in the pathogenesis of T1D (19), and the genome-wide association studies (GWAS) have provided us a clear understanding of risk genes for T1D (20–22). Variations in specific genes, especially the alleles of the human leukocyte antigen (HLA) gene, primarily class II HLA, could increase the risk of T1D (23). Another important factor associated with the pathogenesis of T1D is environmental effects, which could promote the development of T1D through various pathways, including viral infections (24), gut microbiota (25), and diet, etc. (26). Viral infections, especially those that could destroy β cells or activate the immune system, are considered to be the potential environmental triggers for T1D (27). Additionally, studies have demonstrated that there were significant changes in the composition of gut microbiota in patients with T1D (28, 29), which might be related to the development of T1D (30).

In conclusion, the pathogenesis of T1D is very complicated and involves the interaction of autoimmune responses, genetic factors, and environmental factors. Autoimmune responses lead to the destruction of islet β cells, while genetic and environmental factors may increase the risk of developing T1D. A deeper study of these mechanisms could help us better understand the pathophysiology of T1D and then develop more prevention and treatment schemes.

### 2.2 T2D

T2D is a heterogeneous progressive disease (31). Insulin resistance and β cell failure are two main characteristics of T2D, and both of them play crucial roles in disease pathogenesis (32). Insulin resistance refers to a decrease in the metabolic response of target cells to insulin (33). In T2D patients, this means that cells in energy metabolism tissue, including skeletal muscle, the liver, and white adipose tissue, reduce the uptake and processing of glucose, resulting in persistent hyperglycemia (34). For example, skeletal muscle, the largest organ and the primary site for glucose uptake in the human body, was prevented from effectively using insulin, resulting in reduced glycogen synthesis and glucose uptake (35, 36).

**Figure 3:**
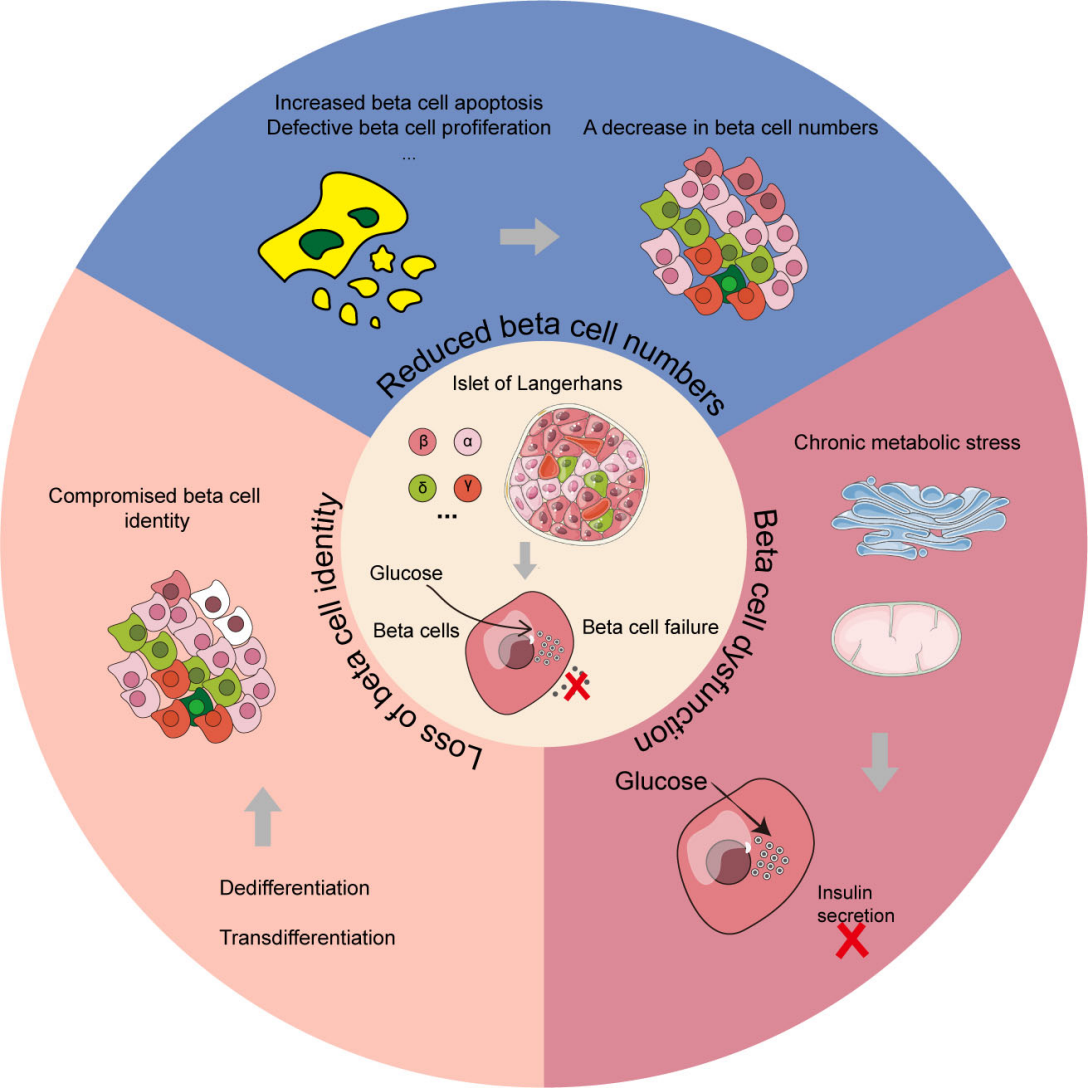
Three main categories of β cell failure in T2D mechanism. The reduction of β cell numbers, β cell dysfunction and loss of β cell identity are the three primary modules for β cell failure mechanism in T2D patients. In the β cell dysfunction module, chronic metabolic stress including ER stress and oxidative stress could lead to the failure of pancreatic β cells. In the loss of β cell identity module, the cells with color of white represent compromised β cells.

The β cell failure is another major indicator of T2D development. Studies have shown that the dysfunction of β cells is a major determinant of the progression from normal glucose to hyperglycemia, and is also central to the development of T2D (37, 38). As discussed by Swisa et al., the potential pathogenesis of β cell failure can be further divided into three main categories (**Figure 3**), which are not mutually exclusive (39). The first type is characterized by a decrease in the number of β cells. According to autopsy research reports of patients with T2D, deficits in β cell mass could be up to 60% compared with body mass index-matched healthy controls (40–42). It is speculated that the mechanism underlying this decrease in β cell mass might be associated with an increase in β cell apoptosis, and attempting to arrest apoptosis may be a potential therapeutic avenue for T2D (43). The second is caused by the dysfunction of β cells arising from chronic metabolic stress conditions, including endoplasmic reticulum (ER) stress and oxidative stress etc. In the progression of T2D, oxidative stress induced by reactive oxygen species (ROS) plays a critical role in the dysfunction of β cells by reducing the secretory capability and promoting apoptosis (44). And reducing the detrimental impacts of ROS on β cells might be a promising approach for preventing the onset and progression of T2D. ER stress may also damage β cells and may even contribute to the development of T2D (45). The inhibition of genes associated with ER stress in β cells might reduce the disease burden for T2D patients. The third type is the loss of β cell identity through dedifferentiation or transdifferentiation into other cell types, which further leads to β cell failure and diabetes progression. Studies using mice with Foxo1-deficient β cells to explore mechanisms of β cell failure in T2D have found that the dedifferentiated β cells in mice were prone to transform into other islet endocrine cell types with different phenotypes, such as α, δ, and PP/γ (46). It suggested that restoring the normal differentiation of β cells might also be an effective therapeutic strategy for diabetes.

## 3 Drug combination therapy for diabetes

### 3.1 Glucose-lowering drugs

The major conventional hypoglycemic drugs used for the treatment of diabetes include diguanides, sulphonylureas, SGLT2 inhibitors, DPP-4 inhibitors, GLP-1 receptor agonists, alpha glucosidase inhibitors, and thiazolidinediones (TZDs), etc. (**Table 1**). These drugs can act on different targets to effectively respond to the complex mechanisms of diabetes, and could work synergistically to better control the blood glucose in diabetes patients.

### 3.2 Drug combination therapy for T1D

In several T1D patients, insulin monotherapy does not achieve the desired effect of blood glucose control, which promoted the analysis of drug combination therapy (**Table 2**).

**Table 2:** The efficacy and safety of commonly used combination therapy in T1D clinical trials.

Combination	HbA1c control	Body weight control	Severe hypoglycaemia^a^	Serious adverse event ^c^
Insulin + SGLTi (47)	Yes	Yes	Yes	Yes
Insulin + Biguanides (48)	No	Yes	Yes	Yes
Insulin + GLP-1 RA (49)	Yes	Yes	No	–
DPP-4i + Vitamin D (50)	Yes	Yes	No	No
Insulin + DPP-4i + PPI + GABA (51)	Yes	Yes	^b^-	No

a Severe hypoglycemia was defined as events characterized by low blood glucose that required the assistance of another person with the administration of carbohydrates, glucagon, or other medical therapies to recover (48). Here is whether the event occurred in the study cohort.

b - represent not mention.

c Serious adverse event was summarized through the results based on different study cohorts. Here, “yes” indicates that in the corresponding study, this combination was reported to have serious adverse events in their cohort.

SGLTi, sodium-glucose cotransporter inhibitors; GLP-1 RA, glucagon-like peptide-1 receptor agonists; DPP-4i, dipeptidyl peptidase-4 inhibitors; PPI, proton pump inhibitors; GABA, γ-aminobutyric acid.

#### 3.2.1 Insulin + SGLT inhibitors

A meta-study of randomized controlled trials (RCTs) has found that there was a remarkable association between the combination therapy of insulin with sodium-glucose cotransporter (SGLT) inhibitors and the decrease in HbA1c of -0.39% (95% CI, -0.43 to -0.36) as well as body weight loss of -3.47% (95% CI, -3.78 to -3.16), suggesting the beneficial effects of this combination in T1D treatment. However, there are some adverse events, including increased incidence rate of genital infection and diabetic ketoacidosis (47). Consistent with above results, in a *post hoc* clinical analysis, the combination of insulin with sotagliflozin (a dual SGLT1/2 inhibitor that could delay glucose absorption and therefore reduce the glucose level of post-prandial (52, 53)) was associated with a significant decrease in HbA1c level, body weight, and a higher diabetic ketoacidosis risk (54). Similarly, studies performed in different populations with T1D have proved that the combination of insulin plus SGLT2 inhibitors (dapagliflozin/empagliflozin) could improve the control of blood glucose and body weight without increasing the risk of hypoglycemia (55–57). Overall, despite adverse events, these studies have revealed that the combination of SGLT inhibitors with basal insulin has beneficial therapeutic effects for T1D patients.

#### 3.2.2 Insulin + biguanides

A clinical trial using overweight adolescents with T1D to assess the effects of metformin added to insulin showed that this combination therapy had no significant improvements in HbA1c control among enrolled participants after six months. Moreover, the incidence of adverse gastrointestinal events was significantly increased by metformin (48). This suggested that the combination of insulin with metformin may not be applicable in overweight adolescents with T1D, and other therapeutic methods to improve the glycemic control of these subjects need to be considered.

#### 3.2.3 Insulin + GLP-1 receptor agonists

The glucagon-like peptide-1 (GLP-1) receptor agonists is a potential medication as insulin-assisted for T1D, with various effects, such as promoting glucose-stimulated insulin secretion and reducing glucagon secretion (58, 59). In a retrospective study using C-peptide-positive patients with T1D, the combination of GLP-1 receptor agonists and insulin treatment could effectively reduce HbA1c from 10.74 ± 0.96% to 7.4 ± 0.58% (*P* < 0.01), and body weight from 71 ± 2.0 to 69 ± 2 kg (*P* = 0.06), which supported the efficacy for this combination therapy (60). Furthermore, the addition of liraglutide (a GLP-1 receptor agonist) to insulin therapy has also demonstrated potential benefits including but not limited to reduced HbA1c level, body weight, and insulin requirement in T1D patients (49, 61–63).

#### 3.2.4 DPP-4 inhibitors + vitamin D

Several pre-clinical experiments have provided support for the synergistic anti-inflammatory and immune regulatory effects of a combination therapy involving vitamin D and dipeptidyl peptidase-4 (DPP-4) inhibitors. This combination therapy has the potential to preserve β cell function in individuals with T1D (64–66). In a retrospective case-control study, it was observed that the combination of vitamin D plus DPP-4 inhibitors could notably prolong the honeymoon phase (partial remission of disease) in newly diagnosed T1D (50). However, further clinical trials are needed to confirm the potential role of this combination therapy in the onset and progression of T1D.

#### 3.2.5 Insulin + DPP-4 inhibitors + PPI + GABA

Proton pump inhibitors (PPI) have the potential to enhance glucose metabolism and insulin secretion (67), and, therefore, the addition of PPI could help to augment the hypoglycemic effects of DPP-4 inhibitors and then to improve T1D control (68). A recent evaluation on the efficacy of combination therapy with γ-aminobutyric acid (GABA, which has regenerative effects on islet β cells (69)), DPP-4 inhibitors, PPI and insulin in T1D patients showed that this combination was associated with significant reductions in fasting blood glucose, HbA1c level, daily insulin dose, and fasting plasma C-peptide etc. After 26-42 weeks of treatments, it has been shown that this combination can improve glycemic control in T1D (51).

#### 3.2.6 Other drug combinations in pre-clinical study

In addition to the above commonly used drug combination strategies in clinical trials, some new combination therapies have also been explored in pre-clinical animal experiments, aiming to provide more potential drug combination treatment options for T1D patients.

For example, it has been reported that the combination therapy of GLP-1 receptor agonists (exendin-4) and PPI (omeprazole) is closely related to the enhanced insulin sensitization actions in streptozotocin (STZ)-induced T1D model mice (70). The study found that triple drug therapy with GABA, DPP-4 inhibitors (sitagliptin), and PPI (omeprazole) has an encouraging therapeutic effect in ameliorating β cell function and managing T1D in non-obese diabetic (NOD) mice (71). In the STZ-induced young and old diabetic mice models, the drug combination therapy consisting of melatonin and DPP-4 inhibitors (sitagliptin) could effectively provoke β cell proliferation in mice, and then ameliorate diabetic manifestations by reducing fasting blood glucose levels, enhancing glucose tolerance, and plasma insulin levels (72). In another study of the diabetic NOD mouse model, co-administration of DPP-4 inhibitors (MK-626) with the histone deacetylase inhibitor (vorinostat) treatment has no notable effect on disease remission, but could increase the area of pancreatic β cells compared with the control group (73). The study of sulphonylureas (glibenclamide) and GABA combined treatment on T1D mouse induced by STZ illustrated that, compared with monotherapy, combination treatment could remarkably improve glycemic control and enhance insulin secretion, which represents a promising anti-diabetic combination scheme (74).

The pre-clinical experiments of these treatments help optimize the choice of drug combinations and could provide decision-making support, thereby facilitating the development of safer and more effective drug combination treatments. However, there still exists a significant gap between pre-clinical experiments and clinical applications, which needs to be evaluated and verified in further clinical trials.

### 3.3 Drug combination therapy for T2D

Similar to T1D, there also exist many drug combination therapies that could efficiently improve blood glucose control in clinical or pre-clinical experiments of T2D (**Table 3**).

**Table 3:** The efficacy and safety of commonly used combination therapy in T2D clinical trails.

Combination	Drug	HbA1c control	Body weight control	Severe hypoglycaemia^a^	Serious adverse event
Biguanides + SU	Metformin+ Glimepiride (75)	Yes	Yes	No	-^b^
Biguanides+ DPP-4i	Metformin+ Vildagliptin (75)	Yes	Yes	No	–
	Metformin+ Sitagliptin (76)	Yes	Yes	No	Yes
	Metformin+ Gemigliptin (77)	Yes	Yes	No	Yes
	Metformin+ Linagliptin (78)	Yes	Yes	No	Yes
	Metformin+ Saxagliptin (79)	Yes	Yes	No	Yes
Biguanides + SGLT2i	Metformin+ Canagliflozin (80)	Yes	Yes	No	Yes
	Metformin+ Dapagliflozin (81)	Yes	Yes	No	Yes
	Metformin+ Empagliflozin (82)	Yes	Yes	No	Yes
Biguanides + GLP-1 RA	Metformin+ Exenatide (83)	Yes	Yes	No	- ^b^
Biguanides + TZDs	Metformin+ Pioglitazone (84)	Yes	No	Yes	Yes
Biguanides + Agi	Metformin+ Acarbose (85)	Yes	Yes	No	Yes
Insulin + SU	Insulin + Glimepiride (86)	Yes	No	No	–
Insulin + DPP-4i	Insulin + Sitagliptin (87)	Yes	Yes	Yes	–
Insulin + SGLT2i	Insulin + Empagliflozin (88)	Yes	Yes	Yes	Yes
Insulin + GLP-1 RA	Insulin + Lixisenatide (89)	Yes	Yes	Yes	–
	Insulin + Semaglutide (90)	Yes	Yes	Yes	Yes
Insulin + TZDs	Insulin + Pioglitazone (91)	Yes	No	Yes	Yes
SGLT2i + DPP-4i	Empagliflozin + Linagliptin (92)	Yes	Yes	No	Yes
DPP-4 i + TZDs	Alogliptin + Pioglitazone (93)	Yes	No	No	Yes
Agi + DPP-4i	Miglitol + Sitagliptin (94)	Yes	–	–	–
SGLT2i + GLP-1 RA	Dapagliflozin + Exenatide (95)	Yes	Yes	–	–

a Severe hypoglycemia was defined as events characterized by low blood glucose that required the assistance of another person with the administration of carbohydrates, glucagon, or other medical therapies to recover (48). Here is whether the event occurred in the study cohort.

b - represent not mention.

c Serious adverse event was summarized through the results based on different study cohorts. Here, “yes” indicates that in the corresponding study, this combination was reported to have serious adverse events in their cohort.

SU, sulphonylureas; DPP-4i, dipeptidyl peptidase-4 inhibitors; SGLTi, sodium-glucose cotransporter inhibitors; GLP-1 RA, glucagon-like peptide-1 receptor agonists; TZDs, thiazolidinediones; Agi, Alpha glucosidase inhibitors.

#### 3.3.1 Biguanides + sulphonylureas

Sulfonylureas, a glucose-lowering drug, could be used as an adjunctive drug for metformin in treatment of T2D (96). In an analysis comparing two different drug combinations, namely sulfonylureas + metformin and DPP4-inhibitors + metformin, the addition of sulfonylureas to metformin demonstrated a higher risk of hypoglycemia and weight gain, indicating that DPP-4 inhibitors may be more suitable than sulfonylureas as adjunctive therapy to metformin for poorly controlled T2D patients (75, 97). In terms of blood glucose control, the combination therapy of sulfonylureas with metformin has shown a similar efficacy of glucose-lowering as other dual combinations, and there was no significant difference in the change of HbA1 among different combinations before and after the treatment (98). However, in other real-world analyses based on different populations, the combination of sulfonylureas with metformin have a higher risk of hypoglycemia events, cardiovascular events, and all-cause mortality compared with other oral anti-diabetic agent added to metformin (99–101). It suggested that with comparable glycemic effects, other oral anti-hyperglycemic agents may be a preferable option as adjunctive to metformin than sulfonylureas.

#### 3.3.2 Biguanides + DPP-4 inhibitors

As discussed above, metformin combinating with DPP-4 inhibitors is a common drug combination treatment in clinical trials with T2D patients. In a 5-year follow-up trial, it was found that newly diagnosed T2D patients who received early combination therapy with metformin and vildagliptin, a DPP-4 inhibitor, had better long-term glycemic control compared to those who only received early monotherapy with metformin (102). In addition, it has been reported that the combination of vildagliptin and metformin has a significant association with HbA1c reduction and body weight loss (103).

An observational study comparing the efficacy of two dual treatments for T2D individuals who did not respond to metformin monotherapy, namely metformin plus sitagliptin (a DPP-4 inhibitor) and metformin plus sulfonylurea, reported that the two combinations had a similar glycemic control effect. However, the combination of metformin and sitagliptin exhibited a lower risk of hypoglycemia and a longer treatment duration. The shorter duration of treatment may be attributed to some factors, such as inadequate effectiveness, severe hypoglycemia or other serious side effects (104). According to reports, the combination therapy of metformin and sitagliptin has also shown a significant improvement in blood glucose levels in patients with T2D after hospital discharge (105). Moreover, a real-world study indicated that the initial combination therapy of metformin with sitagliptin exhibited a consistent and prolonged glycemic improvement for a period of up to 4 years (106), supporting the long-term effectiveness of this combination therapy. And the function of pancreatic β cells in T2D patients could also be notably improved within 24 hours after receiving the combination of metformin with sitagliptin treatment (107).

In a randomized controlled trial, a new DPP-4 inhibitor called gemigliptin was added to metformin for the initial treatment of T2D patients. The findings revealed that combination therapy exhibited significant superiority in glycemic control without any severe adverse effects compared with the monotherapy groups of either metformin or gemigliptin. Specifically, the average change in HbA1c levels from baseline to week 24 was -2.06% in the combination therapy group, -1.24% in the gemigliptin monotherapy group, and -1.47% in the metformin monotherapy group (77). In another randomized controlled study, compared with the metformin-glimepiride combination (metformin- sulphonylureas), the combination of metformin-gemigliptin (metformin-DPP-4 inhibitor) achieved more effective glycemic control in T2D patients without increasing the risk of hypoglycemia or weight gain, which may be related to improvements in gut microbiota (108).

A study on the addition of metformin to linagliptin (a DPP-4 inhibitor) demonstrated that this combination exhibited good tolerability for blood glucose control in newly diagnosed T2D patients and was not influenced by baseline factors such as HbA1c, age, and race (78). In addition, the dual combination therapy of linagliptin and metformin has been reported to achieve satisfactory blood glucose control of T2D individuals in different countries (109–113), indicating the feasibility and good tolerability of this combination treatment in clinical practice for T2D patients. It could serve as an effective strategy for glycemic improvement, particularly in newly diagnosed T2D patients. More importantly, the low incidence of hypoglycemic events, weight gain, and other serious side effects further increase the appeal of this combination treatment (114, 115), which could provide essential insights for doctors and patients to develop personalized treatment plans.

Saxagliptin, a DPP-4 inhibitor, was developed for T2D treatment (116). In a phase III clinical trial to evaluate the efficacy and safety of the saxagliptin-metformin combination, researchers found that the combination treatment showed better tolerability compared to the monotherapy of saxagliptin or metformin, and the duration of blood glucose control in the combination therapy could be up to 76 weeks (117). Similarly, several clinical trials with different populations have reported the pronounced benefits of saxagliptin-metformin combination therapy, including significant improvements in blood glucose, well tolerability, low risk of hypoglycemia, and serious adverse events (118–122).

Overall, the results of these studies consistently demonstrated the favorable clinical benefits of combined therapy with biguanides and DPP-4 inhibitors. The synergistic interaction between these two drugs could lead to improved blood glucose control and offer an effective treatment option for patients with T2D.

#### 3.3.3 Biguanides + SGLT2 inhibitors

SGLT2 inhibitors, which are responsible for most glucose reabsorption in the kidneys, play a crucial role in blood glucose regulation for individuals with diabetes (123). Common SGLT2 inhibitor drugs include canagliflozin, dapagliflozin, empagliflozin, janagliflozin, etc.

A double-blind phase III clinical trial evaluated the efficacy of the initial therapy with canagliflozin and metformin in drug-naïve patients with T2D. The combination therapy group demonstrated statistically significant improvements in glycemic control and body weight reduction when compared with canagliflozin or metformin monotherapy groups (80). This suggested that the combination therapy of metformin-canagliflozin showed more potent effectiveness and tolerance than monotherapy. In some dual therapy trials with subjects already receiving metformin monotherapy and then being assigned to receive canagliflozin, the combination of canagliflozin and metformin also showed significant control of blood glucose, body weight, and systolic blood pressure in T2D patients (124–126). In T2D patients who were inadequately controlled with metformin alone, researchers found that, compared with placebo, the combination of dapagliflozin-metformin treatment resulted in a notable control of HbA1c, fasting blood glucose, and body weight from baseline, and this treatment did not increase the risk of hypoglycemia (81, 127–131). The combination therapy of empagliflozin with metformin in the treatment of individuals with T2D also demonstrated superior improvements in glycemic control, weight loss, and blood pressure management, with favorable tolerability (82, 132–134). Considering the promising potential of this combination in controlling blood pressure and body weight, some scholars have suggested that it may offer a feasible therapeutic option with significant improvements for T2D patients who have suboptimal glycemic control on metformin alone, particularly those who would benefit from a modest decrease in body weight and blood pressure or have risk factors for declining renal function and cardiovascular disease (135, 136).

The results of these different clinical trials all pointed to the fact that the combinations of metformin and different SGLT2 inhibitor drugs have an encouraging efficacy in the management of T2D, including better glycemic improvements, weight loss, blood pressure control, etc. In addition to these advantages, the combination of metformin-SGLT2 inhibitors also showed potential benefits in cardiovascular and renal protection, providing comprehensive protection for patients with T2D (135, 137). In summary, the combinations of metformin and SGLT2 inhibitors have demonstrated desirable effectiveness in the management of T2D. However, the selection and adjustment of treatment plans should still be personalized based on the characteristics of individual patients and the guidance of healthcare professionals.

#### 3.3.4 Biguanides + GLP-1 receptor agonists

GLP-1 receptor agonists (GLP-1RAs), such as lixisenatide, exenatide, liraglutide, and semaglutide, have been widely utilized in the therapy of T2D due to their advantages in blood glucose control, weight loss, and protection of pancreatic β cells, etc (138, 139). In studies evaluating the impact of the combination therapy with exenatide and metformin on patients with T2D, it was found that compared with the placebo-metformin group, the group of exenatide-metformin showed better glucose and body weight control (83). This combination also played a critical role in protecting β cells and reducing inflammation (140). In addition, the combination of exenatide and metformin has been reported to have the potential in enhancing certain adipocytokine levels, improving insulin sensitivity in T2D patients (141), and reducing insulin resistance (142). Interestingly, researchers have observed notable variations in the therapeutic efficacy of the exenatide-metformin combination therapy between different genders, with female patients displaying a more favorable treatment outcome for decreasing the levels of HbA1c, BMI, improving insulin sensitivity, reducing insulin resistance (*P*<0.05), etc. It suggested that this combined method may be more suitable for female individuals with T2D (143).

#### 3.3.5 Biguanides + TZDs

Thiazolidinediones (TZDs), also known as glitazones, are a class of insulin sensitizers primarily used for the treatment of T2D (144). They are responsible for regulating insulin sensitivity, helping cells better respond to insulin and effectively utilize glucose (145). According to reports, TZDs mainly modulated peroxisome proliferators activated receptors (PPAR) in adipose tissue, gradually increasing cell receptiveness to insulin (146). There are two main TZDs, namely rosiglitazone and pioglitazone (147). In a parallel-controlled study evaluating the efficacy of fixed-dose combination therapy with pioglitazone and metformin in treating T2D patients, it was found that the combined therapy group showed the greatest and statistically significant reduction in average HbA1c compared to monotherapy (84). Although slight weight gain was observed in the combination therapy group, considering the significant improvement in blood glucose levels, the researchers still concluded that this combination therapy is effective and well-tolerated for T2D patients (148).

#### 3.3.6 Biguanides + alpha glucosidase inhibitors

A study comparing the effects of the combination of acarbose (alpha glucosidase inhibitors) and metformin therapy with acarbose monotherapy in the treatment of T2D reported that the combination treatment could greatly improve glycemic control in T2D individuals with a significant reduction in HbA1c, fasting plasma glucose, and postprandial glucose compared to baseline (*p* < 0.0001). Additionally, the combination therapy was superior to monotherapy in weight control without increasing the hypoglycemic risk (85). These findings indicated that this combined therapy could provide encouraging advantages in managing T2D.

#### 3.3.7 Insulin + sulphonylureas

For many poorly controlled T2D patients who only received insulin monotherapy, clinical doctors may also recommend they to use some oral anti-diabetic medications at the same time. In a retrospective study, researchers found that the combination of insulin and oral glimepiride (sulphonylureas) in patients with T2D showed a more significant improvement in HbA1c control compared to insulin alone, decreasing from 8.5 +/- 0.6% to 7.4 +/- 0.8% (*P* < 0.0001). Additionally, the required insulin dosage was also reduced markedly (86). Therefore, it is concluded that adding glimepiride to insulin therapy could sustainably improve blood glucose control in T2D patients.

In another clinical trial, patients with poor insulin control were randomly assigned to either the addition of glimepiride or insulin increase. Compared with insulin therapy alone, the insulin-glimepiride combination also showed a significant improvement in blood glucose control and a notable decrease in insulin dosage. Interestingly, researchers have observed a negative correlation between changes in HbA1c and changes in serum high molecular weight (HMW) adiponectin levels in the combination therapy group, suggesting that regulating changes in serum HMW adiponectin levels may contribute to viable improvements in blood glucose control (149).

#### 3.3.8 Insulin + DPP-4 inhibitors

When Sitagliptin, a DPP-4 inhibitor, was added to insulin therapy in the treatment of poorly controlled patients with T2D, it was found that the combined group had a greater reduction in HbA1c level compared to the group with increased insulin dose. Moreover, there were fewer adverse effects in the combined group, including lower frequency and severity of hypoglycemic events, as well as less weight gain (87).

#### 3.3.9 Insulin + SGLT2 inhibitors

In a 78-week clinical trial for assessing the efficacy and safety of adding empagliflozin to basal insulin therapy in patients with poorly controlled T2D, individuals were randomly assigned to different doses of empagliflozin (10mg or 25 mg) or a placebo group. Results indicated that the addition of empagliflozin could significantly improve blood glucose management in T2D patients with inadequate basal insulin control. This combination therapy not only reduced HbA1c levels and insulin dosages, but also contributed to weight control and systolic blood pressure reduction (88). These beneficial effects could provide T2D patients with a satisfactory option for antihyperglycemic treatment. However, when implementing this combination treatment approach, careful monitoring of practical conditions and drug safety is still essential to ensure optimal clinical outcomes and the well-being of patients.

#### 3.3.10 Insulin + GLP-1 receptor agonists

A meta-analysis exploring the efficacy of combining lixisenatide with basal insulin revealed that, compared with insulin monotherapy, this combination treatment could effectively reduce HbA1c levels, particularly in controlling postprandial glucose (PPG). However, there were no notable differences in fasting plasma glucose (FPG) levels between these two groups. Insulin could effectively control FPG, but further oral management is required for optimal control. Therefore, it suggested that T2D patients with suboptimal glycemic control on basal insulin may benefit from the addition of lixisenatide to further enhance their treatment effectiveness (89).

In a clinical trial involving diverse populations from different countries and regions, it was reported that compared with the placebo group, the combination of semaglutide, another GLP-1 receptor agonist, with basal insulin had outstanding advantages in anti-diabetic management, including more pronounced reductions of HbA1c and body weight (90). These positive findings suggested that adding semaglutide to basal insulin therapy is an effective strategy to improve blood glucose control and body weight control in patients with T2D. Continuing to promote the research and application of this combination therapy was expected to offer more effective treatment options for T2D patients worldwide. However, when adopting this treatment, individual differences and potential side effects need to be carefully considered to ensure the optimal clinical benefit and patient safety.

#### 3.3.11 Insulin + TZDs

In many clinical trials, insulin combined with pioglitazone (TZDs) has been widely utilized to treat T2D patients. For example, in a study exploring the benefits of insulin plus pioglitazone in poorly controlled T2D patients, this combination therapy not only improved blood glucose levels and reduced daily insulin dosages, but also had a positive effect on lipid level control, suggesting that the co-administration of insulin and pioglitazone could improve the therapeutic effects of diabetes patients in multiple aspects (91).

#### 3.3.12 SGLT2 inhibitors + DPP-4 inhibitors

It was reported that the combination of empagliflozin (SGLT2 inhibitors) and linagliptin (a DPP-4 inhibitor) could provide more effective hypoglycemic effects than monotherapy in patients who did not respond to metformin, with a lower risk of hypoglycemia (92). This suggested that, for patients with poorly controlled by metformin, dual therapy with empagliflozin and linagliptin or a triple combination of empagliflozin, linagliptin and metformin may be a viable treatment option.

#### 3.3.13 DPP-4 inhibitors + TZDs

In a clinical trial evaluating the effect of a combination of alogliptin (a DPP-4 inhibitor) and pioglitazone (a TZD) in treating drug-naïve patients with T2D, the combination treatment showed more significant reductions in HbA1c levels and fasting blood glucose levels than monotherapy alone. Moreover, the safety of the combination therapy was consistent with that of monotherapy. Compared with baseline, the HbA1c decreased by an average of -1.7 ± 0.1% in the combination treatment group, while the changes in alogliptin monotherapy and pioglitazone monotherapy were -1.0 ± 0.1% and -1.2 ± 0.1%, respectively, showing the remarkable efficacy in the combination treatment group (93).

#### 3.3.14 Other drug combination options

In addition to the combination of certain medications discussed above, there are numerous other dual-combination approaches for the treatment of T2D, including combinations of alpha glucosidase inhibitors and DPP-4 inhibitors (94), SGLT2 inhibitors and GLP-1 receptor agonists (95), etc. These combined medication strategies could help to further lower blood glucose levels and achieve better diabetes management and treatment. Furthermore, multi-drug combination therapy involving three or more medications has also emerged in various clinical trials (150–154). Common multi-drug therapies include combinations of different classes of medications, such as oral hypoglycemic agents or insulin. This comprehensive treatment strategy of multiple drugs combination could address the complex physiological processes of diabetes and help patients achieve more stable glycemic control and management.

## 4 Drug combination therapy for diabetic complications

Long-term hyperglycemia in diabetic patients can lead to various complications of varying severity. In clinical trials, researchers have explored various combinations of medications for the treatment of different complications, aiming to achieve personalized management and treatment for diabetes patients. Here, we briefly discussed the benefits and risks of drug combination therapies for two common complications: diabetic nephropathy and diabetic fatty liver.

### 4.1 Drug combination therapy for diabetic nephropathy

Diabetic nephropathy (DN) is one of the most common complications of diabetes, and around 40% of diabetes patients will have it (155). It is the major cause of chronic kidney failure and end-stage kidney disease (ESKD) (156). DN is characterized by the accumulation of extracellular matrix in the glomeruli and tubulointerstitium, as well as the thickening of the glomerular basement membrane (9). These changes could gradually destroy the glomeruli and renal tubules, resulting in proteinuria, hypertension, and declining kidney function, etc (157). The primary strategy for treating DN is to strictly control blood glucose and blood pressure (158). However, when diabetic nephropathy progresses to a certain stage, simple control of blood glucose and blood pressure may be insufficient to control the progression of the disease. In such cases, the combination use of medications might be a feasible treatment direction.

#### 4.1.1 SGLT2 inhibitors + others

In a study of diabetic model mice induced by STZ and high-fat diet, researchers have found that the co-administration of empagliflozin (SGLT2 inhibitors) with ursolic acid could help to better improve glucose control and alleviate or counteract the side effects associated with each other compared with monotherapy of empagliflozin or ursolic acid alone. More importantly, this combination was proved to have positive effects in mitigating diabetic nephropathy by reducing inflammation, oxidative stress and renal fibrosis through inhibition of the TGF-β/SMAD/MAPK signaling pathway (159). These encouraging results suggested that the combination of empagliflozin-ursolic acid may be a potentially effective approach for treating DN and provide a solid foundation for future clinical analysis of DN. In another pre-clinical study with diabetic mice induced by STZ, it was reported that the combination of dapagliflozin (SGLT2 inhibitors) with irbesartan (an angiotensin II receptor blocker, ARB) has potential benefits in the protection of renal function and structure with reduced albuminuria, improved renal function parameters and renal histopathological changes etc (160). Due to the complementary mechanisms of SGLT2 inhibitors and ARBs on kidney, this drug combination could provide more effective renal protection and may represent a viable management option for DN. The combination of empagliflozin (an SGLT2 inhibitor) with linagliptin (a DPP-4 inhibitor) was also reported to have higher benefits of renal protection than the single use of SGLT2 inhibitors in diabetic mice (161).

#### 4.1.2 ACE inhibitors + others

Angiotensin converting enzyme (ACE) inhibitors are a class of drugs that can effectively reduce the systemic vascular resistance of patients with chronic kidney disease, thereby achieving long-term renal protection (162). Common ACE inhibitors include lisinopril, benazepril and fosinopril etc.

A recent study aiming to assess the efficacy of the lisinopril-naringenin combination in DN has found that the combination could significantly improve the biochemical and urine parameters of diabetic mice induced by STZ. Moreover, combination therapy can also help alleviate renal oxidative stress and renal damage, which indicates the beneficial effects of this combination on DN mouse (163). According to reports, benazepril combined with leflunomide was more effective in improving renal function and alleviating renal damage in DN mice than the use of either drug alone (164).

#### 4.1.3 ARBs + others

Angiotensin II receptor blockers (ARBs), such as telmisartan, candesartan, losartan, and valsartan, are a class of drugs that can significantly reduce the risk of end stage renal disease. As discussed above, ARBs would be used in combination with other medications for the treatment of DN mice. In an experimental study on the use of an STZ-induced diabetic mouse model, the combination of telmisartan and sildenafil exhibited better amelioration of DN than a single drug alone with a notable decrease in proteinuria, blood urea nitrogen and an increase in superoxide dismutase, etc (165). Similarly, the dual therapy of candesartan with spironolactone can improve the renal function parameters, including reduced serum levels of urea and creatinine in diabetic mice. Additionally, compared with monotherapy, combination therapy can provide additional renal protection presumably by weakening inflammatory response and oxidative status markers (166). In a diabetic mouse model, the combination of losartan and nitro-oleic acid could result in a reduction of albuminuria, restoration of glomerular filtration barrier structure, attenuation of glomerulosclerosis, and inhibition of renal oxidative stress and inflammation, indicating that there is great potential to reverse kidney injury in DN (167). In another clinically relevant animal model of early DN, compared with monotherapy with valsartan (ARBs), the valsartan-sacubitril treatment showed superior efficacy in reducing proteinuria, preserving renal ultrastructure, and mitigating tubular injury. Importantly, despite the lack of significant improvements in blood pressure, blood glucose, and oxidative stress, the combined treatment exhibited pronounced renal protective effects. This unique advantage of combination therapy provides independent evidence for its efficacy, which is not reliant on conventional treatment factors (168).

### 4.2 Drug combination therapy for fatty liver

Hyperglycemia could also affect the liver, leading to liver tissue damage and inflammatory reactions in diabetic individuals (169). It was reported that non-alcoholic fatty liver disease (NAFLD) occurred in 50-60% of individuals with T2D and up to 45% of T1D patients (170, 171).

#### 4.2.1 GLP-1 receptor agonists + SGLT2 inhibitors

In a *post hoc* study using 695 T2D patients inadequately controlled by metformin alone, exenatide (GLP-1 receptor agonists) and dapagliflozin (SGLT2 inhibitors) combination could notably reduce the fatty liver index and the fibrosis-4 index from baseline, suggesting the beneficial effects in improving markers of hepatic steatosis and fibrosis (172). This evidence confirmed that the combination of GLP-1 receptor agonists and SGLT2 inhibitors could provide additional liver protection for patients with T2D.

#### 4.2.2 Statins + biguanides

Statins are medications that can inhibit the hydroxymethylglutaryl-CoA (HMG-CoA) reductase enzyme, and are highly effective in reducing cholesterol levels (173). Common statins medications include atorvastatin, simvastatin, and rosuvastatin, etc. In a pre-clinical study, T2D rats were fed with a high-fat diet, which exacerbated the markers of hepatic injury. The combination of atorvastatin (statins) and metformin could significantly decrease systemic and hepatic oxidative stress, portal inflammation, C-reactive protein, adiponectin, and interleukin-6 etc (174). These suggested that the atorvastatin-metformin has a significant therapeutic effect in ameliorating liver injury in T2D with hyperlipidemia.

#### 4.2.3 Statins + insulin

Similarly, the combination of atorvastatin and insulin has shown impressive blood glucose and lipid control in diabetic rats fed with high-fat diet. It has also shown the improvement in its protective effects on liver inflammation and oxidative stress (175). These results indicated that atorvastatin-insulin therapy can provide liver protection for T2D patients with hyperlipidemia and can further contribute to overall improvements in patient health.

#### 4.2.4 Statins + cholesterol absorption inhibitors

In a study involving 19 patients with T2D and NAFLD, researchers found that, after 6 months of treatment with the combination of simvastatin (statins) and ezetimibe (a cholesterol absorption inhibitor), significant reductions were observed in serum alanine aminotransferase (ALT), aspartate aminotransferase (AST), low-density lipoprotein (LDL), and other markers, which suggested that this combination therapy is an effective treatment approach for patients with T2D and NAFLD (176).

#### 4.2.5 SGLT2 inhibitors + cholesterol absorption inhibitors

In a study on the population of diabetic individuals in Korea, researchers found that individuals treated with empagliflozin (SGLT2 inhibitors) and/or ezetimibe (a cholesterol absorption inhibitor) had a significantly lower risk of developing fatty liver than those in the untreated reference group. The odds ratio (OR) was 0.962 (95% CI: 0.936-0.989) for the ezetimibe therapy group, 0.527 (95% CI: 0.454-0.611) for the empagliflozin therapy group, and 0.509 (95% CI: 0.362-0.714) for the ezetimibe plus empagliflozin group, compared with the reference group (177). The combination therapy was more effective than monotherapy in reducing the incidence of fatty liver, which highlighted the superiority of this combined regimen in the prevention of fatty liver.

## 5 Discussion and conclusion

Diabetes is a heterogeneous metabolic disorder with complex pathogenesis and individual differences, thus, monotherapy may fail to meet the needs of all patients in blood glucose control or complication prevention. Drug combination therapy could contribute to desired glucose control and improve pancreatic function through various mechanisms and effects. For patients who are unresponsive or poorly controlled with monotherapy, those who may experience adverse reactions to a single medication, or those with concurrent conditions such as hypertension or hyperlipidemia, combination therapy may be a viable treatment approach, which could provide more comprehensive and personalized treatment strategies for diabetes and diabetic complications. However, it is crucial to note that drug combination therapy may not be suitable for all patients. Individuals with a history of severe adverse reactions to specific medications, those with contraindications to certain drug combinations, or patients who prefer or adhere better to simpler treatment regimens may not be ideal candidates for combination therapy. Careful consideration of individual patient characteristics and preferences is essential in determining the appropriateness of drug combination therapy for diabetic management.

In this review, we primarily summarized the benefits and risks of combination drug therapy in the management of diabetes based on diverse clinical trial results. However, we also acknowledge certain limitations in existing clinical studies. Firstly, the sample sizes in the majority of clinical studies are relatively small, and they may not adequately represent the target population. This could potentially impact the generalizability and extrapolation of study results. To gain a more comprehensive understanding of the effects of combination drug therapy, future research may need to consider expanding sample sizes and ensuring the representativeness of samples to better reflect real-world clinical scenarios. Secondly, the homogeneity of study populations is another notable limitation. An ideal study design should encompass individuals from different ethnicities and geographical locations, rather than relying on homogeneous populations. Despite these limitations, we maintain an optimistic outlook on the potential of combination drug therapy in the management of diabetes. In the future, we look forward to seeing additional high-quality and diversified research efforts that will further deepen our understanding of the role of combination drug therapy in diabetic management.

Compared with monotherapy, drug combination therapy has several pronounced advantages: firstly, different drugs can act on multiple targets, and synergistically regulate diabetes through various mechanisms and pathways, which may compensate for the limitations of single-drug therapy and help patients achieve better blood glucose control. Secondly, by combining multiple drugs, it is possible to reduce the dosage of individual drugs, thereby minimizing the toxic side effects and adverse reactions associated with medication. This helps patients better tolerate the treatment and improves the long-term compliance of treatment. Additionally, combination therapy is also beneficial to reduce the risk of diabetic complications in patients. Good glycemic control can effectively prevent and/or delay the development of diabetes-associated complications. Combination therapy, through various pathways, could efficiently control blood sugar levels, and thus reduce various complications associated with hyperglycemia. Finally, combination therapy can comprehensively incorporate different drug combinations based on the specific conditions, needs, and characteristics of the patient, providing more personalized treatment strategies, and ultimately enhancing the overall health status of patients.

However, the combination drug therapy strategy also brings certain challenges that should not be ignored. For example, the simultaneous use of three or more drugs at the same time undoubtedly increases the complexity of the treatment regimen. Patients need to manage multiple drugs according to specific instructions, dosage, and medication time, which might easily lead to confusion or omissions, seriously affecting the effectiveness of treatment. Furthermore, many drugs may interact with each other, resulting in changes or reductions in drug efficacy, or even increased drug toxicity. From a cost perspective, drug combination therapy requires the use of multiple different drugs simultaneously, which increases the financial burden of treatment, especially for patients who cannot afford expensive drugs.

Despite some limitations, we hold optimistic attitudes about the prospects of combined drug therapy for diabetes and its complications based on existing clinical studies. Additionally, as technology advances, combined drug therapy is making significant progress toward personalized treatment. Genomic exploration of individual genetic information provides a strong foundation for personalized care. Through the integration of emerging biomarkers and analysis of clinical data, we can acquire more comprehensive patient information, facilitating improved adjustments to treatment plans tailored to individual patient needs. As more clinical trials and pre-clinical experiments continue, we believe that more reliable and stable strategies for combined drug therapy will be developed. Importantly, based on the rich data resources generated experimentally, researchers have developed various computational methods for drug combination prediction. Machine learning methods, deep learning in particular, have been successfully applied to prediction of drug interactions (178, 179). For example, a recent study demonstrated that the prediction framework called DeepIDC, based on heterogeneous information and deep learning, can accurately predict potential interactions of injections and efficiently narrow down the search space for drug combinations, providing rational recommendations for clinical pharmacists (180). Additionally, extensive research has been conducted to accelerate the identification process of novel potential combined drug therapies using attention mechanism models (181–183). Attention mechanism, an essential part of deep learning, can capture the relationships and interaction patterns between drugs in drug combination prediction models (184). They enable the models to selectively focus on relevant information for the target task, thereby improving the performance and efficiency of the models. Through computational methods, we can predict the probability of interactions between different drugs, aiding in the identification of potential effective drug combinations. Additionally, computational strategies play a crucial role in tailoring personalized treatments, thereby enhancing the effectiveness of treatments. It should be noted that further research is needed to evaluate the long-term effects, safety, cost-effectiveness, and adaptability to different populations before these combined strategies can be applied in clinical practice.

In the future, we anticipate further exploration of clinical research on drug combination therapy, specifically emphasizing the long-term effectiveness and safety of medication combinations. Leveraging computational methods and additional genetic information as supportive tools, we believe drug combination therapy will progress toward a more personalized approach. Considering the heterogeneity of diabetes, we hope to explore drug combination therapy based on finer subpopulations within diabetes, which will contribute to a more comprehensive understanding of individual responses to treatment, offering guidance for more effective clinical management.

## Author contributions

XX: Data curation, Visualization, Writing – original draft. CW: Investigation, Methodology, Visualization, Writing – original draft. YH: Investigation, Methodology, Visualization, Writing – original draft. TW: Investigation, Writing – original draft. YY: Investigation, Writing – original draft. PC: Investigation, Methodology, Visualization, Writing – original draft. YZ: Investigation, Methodology, Visualization, Writing – original draft. JH: Investigation, Writing – original draft. KD: Conceptualization, Validation, Writing – review & editing. DY: Funding acquisition, Project administration, Validation, Writing – review & editing. HL: Conceptualization, Funding acquisition, Project administration, Supervision, Writing – review & editing.
